# Pixelwise Estimation of Signal-Dependent Image Noise Using Deep Residual Learning

**DOI:** 10.1155/2019/4970508

**Published:** 2019-09-09

**Authors:** Hanlin Tan, Huaxin Xiao, Shiming Lai, Yu Liu, Maojun Zhang

**Affiliations:** College of System Engineering, National University of Defense Technology, Changsha 410073, China

## Abstract

In traditional image denoising, noise level is an important scalar parameter which decides how much the input noisy image should be smoothed. Existing noise estimation methods often assume that the noise level is constant at every pixel. However, real-world noise is signal dependent, or the noise level is not constant over the whole image. In this paper, we attempt to estimate the precise and pixelwise noise level instead of a simple global scalar. To the best of our knowledge, this is the first work on the problem. Particularly, we propose a deep convolutional neural network named “deep residual noise estimator” (DRNE) for pixelwise noise-level estimation. We carefully design the architecture of the DRNE, which consists of a stack of customized residual blocks without any pooling or interpolation operation. The proposed DRNE formulates the process of noise estimation as pixel-to-pixel prediction. The experimental results show that the DRNE can achieve better performance on nonhomogeneous noise estimation than state-of-the-art methods. In addition, the DRNE can bring denoising performance gains in removing signal-dependent Gaussian noise when working with recent deep learning denoising methods.

## 1. Introduction

Noise level is an important parameter which decides how much the input noisy image should be smoothed. This parameter is directly required for many well-known denoising algorithms including Wiener filtering [1], nonlocal means (NLM) [2], BM3D [3], sparse representation-based denoising [4], and deep learning denoising methods [5, 6].

Image denoising is often formulated to remove Gaussian white noise, which is additive and homogeneous. *Homogeneous* means that the noise variance is a constant for all pixels over the input noisy image and does not change over the position or color intensity of a pixel [7]. This formulation significantly simplifies the process of image denoising, where noise level is the only parameter required to model noise.

**Figure 1:**
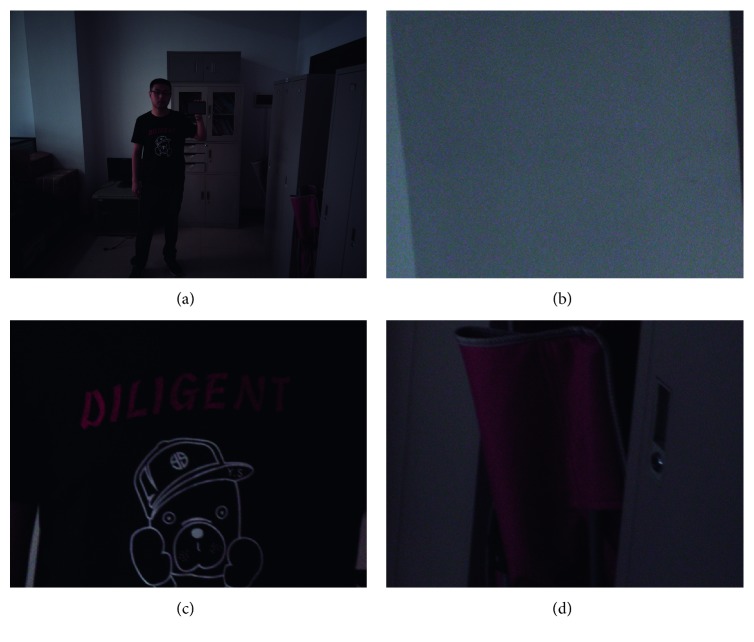
Signal-dependent image noise. (a) Linear RGB image processed from a raw image without denoising, brightening, or gamma correction. (b) Bright part (strong noise). (c) Dark part (slight noise). (d) Normal part (medium noise).

However, homogeneous noise assumption is not valid in real-world images [8].  Figure 1 illustrates a linear RGB image without denoising or gamma correction. We can see that noise in the bright part is stronger than noise in the dark part in the linear RGB image (“bright” and “dark” here are common concepts without strict mathematical definition; we just use the two words to describe the phenomenon). Previous works [9, 10] have pointed out that noise in raw images or linear RGB images consists of two parts: (1) read noise, which is Gaussian and independent of the signal, and (2) shot noise, which is Poisson with variance equal to the signal level. Therefore, real image noise can be well modeled by a signal-dependent Gaussian distribution:

(1) $$\begin{array}{c} x_{p}∼N\left(y_{p},\sigma_{r}^{2}+\sigma_{s}y_{p}\right), \end{array}$$

where *x*_*p*_ is a true noisy measurement and *y*_*p*_ is the true intensity. The noise parameters *σ*_*r*_ and *σ*_*s*_ are fixed but can vary across images as sensor gain changes [10].

Another observation is that noise in the dark part seems stronger than that in the bright part in standard RGB (sRGB) images. The reason is that sRGB images are gamma corrected and that the intensity including noise in the dark part is amplified more times than that in the bright part.

**Figure 2:**
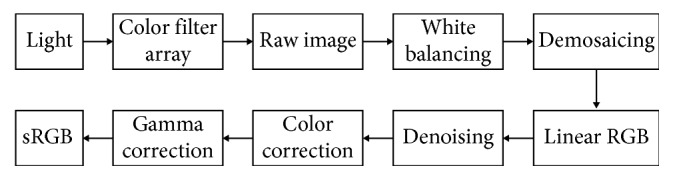
Image processing pipeline (ISP). In the ISP, light signals are converted to the raw image through a color filter array (CFA) and then go through white balancing and demosaicing to result in a linear RGB image. This linear RGB image further goes through denoising, color space conversion, and finally nonlinear gamma correction to produce the final sRGB image.

In the image signal processing (ISP) pipeline, as shown in Figure 2, denoising is performed on linear RGB images before gamma correction. Hence, the signal-dependent model equation (1) is a more precise model in industry than the commonly used signal-independent Gaussian model, and performance gain under the model is of importance.

Since the image noise variance is not constant in an image, granularity of noise-level estimation needs to be smaller in order to improve denoising performance. Pixelwise noise-level estimation is the ultimate form.

Traditional denoising methods may require some modifications to take advantages of pixelwise noise estimation. However, deep learning methods use the pixelwise noise estimation results naturally. Recent deep learning-based image denoising methods [5, 6] construct a noise-level map to introduce noise-level information to denoising networks. Currently, the noise-level map is filled with the same scalar. However, the noise-level map can also be constructed with pixelwise noise-level estimation. Our experiments will show that using pixelwise noise-level estimation leads to better performance on nonhomogeneous noise removal.

In this paper, we propose a deep residual convolutional neural network for pixelwise noise-level estimation. The main architecture simply consists of a stack of customized residual blocks, which is more proper for noise estimation. No pooling operation or a larger stride of convolution is adopted in the proposed architecture, which always intends to extract high-level features, such as semantic information. However, such a refined feature is unnecessary for noise estimation, which always focuses on low-level features, e.g., boundary and local variance. Given a noisy image, the proposed method is able to produce a pixelwise noise-level estimation map as well as a global scalar noise level. The contributions of this study are as follows:

1. Although many works have pointed out that noise standard deviation is not uniform across the image [8, 9], this paper is the first work to give a pixelwise Gaussian noise-level estimation map. The method works on a more precise signal-dependent noise model (1). Moreover, the pixelwise estimation map can also collaborate with recent deep learning-based denoising methods [5], which require a noise-level estimation map as the input.
2. Deep convolutional neural networks are adopted to provide pixel-to-pixel predictions, which are carefully designed by several residual blocks.
3. In terms of traditional scalar average estimation error, the proposed method is able to compete with the state-of-the-art methods [7] in both traditional homogeneous scalar noise estimation and nonhomogeneous noise estimation.

## 2. Related Works

### 2.1. Noise-Level Estimation

The challenge of noise-level estimation lies in distinguishing high-frequency noise from high-frequency image details. To overcome the difficulty, traditional methods often divide an image into fixed-size patches and search for flat parts to estimate the noise level [11–14]. They assume that there are sufficient numbers of flat areas in the processed image [15–18].

Lee and Hoppel [19] treat smallest local variance patches as selected flat patches. Shin et al. [12] choose the patches whose standard deviation in intensity is close to the minimum one within all patches. Then, the processed image is convolved with filters (e.g., Laplacian) to remove the influence of image content [20]. The second-order nature of the Laplacian tends to make it sensitive to noise while suppressing smoothly varying and uniform regions and enhancing edges. After filtering, the image still consists of both noise and object edges. Therefore, some object edge detectors are proposed to recognize and remove edges. Finally, robust statistical methods such as the median of local estimates [20], the mode of local estimates [21], and the average of several smallest local estimates [22, 23] are applied to estimate the final noise variance.

In recent years, PCA-based approaches [7, 24, 25] have attracted great attention since they can successfully deal with scenes containing rich textures. These methods are based on the observation that patches taken from the noiseless image often lie in a low-dimensional subspace, instead of being uniformly distributed across the ambient space. In [24], the authors show that the noise variance can be estimated as the smallest eigenvalue of the image block covariance matrix. In [25], the authors prove that the naive PCA-based approach cannot lead to the satisfactory noise estimation, especially in complex scenes. To this end, their approach adds a process of selecting low-rank patches based on the gradients and other statistics of the patches. In [7], the authors estimate the statistical relationship between the noise level and the eigenvalues of the covariance matrix of patches. Also, they observe that the eigenvalues almost follow a Gaussian distribution in redundant dimensions. As a result, the work reported in [7] achieves the state-of-the-art performance.

All methods above compute a scalar estimation, which works well with traditional denoising methods including BM3D [3] and NLM [2]. However, those methods all simplify the noise with homogeneous Gaussian variance, which is not in accordance with the real noise model (equation (1)).

Recent works on color image noise estimation consider noise variance difference on different color channels [26], which estimate three noise variances or a covariance matrix for an image. This estimation in cooperation with certain denoising methods [27] can improve denoising performance on removing multivariate Gaussian noise.

### 2.2. Deep Learning Denoising Methods

**Figure 3:**
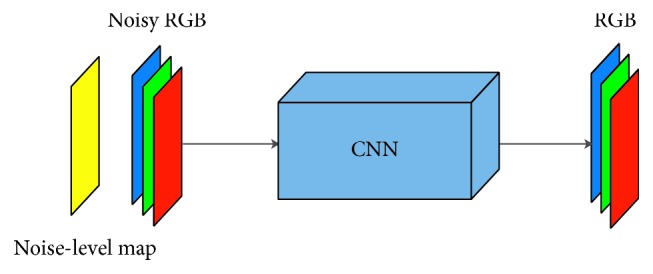
Deep denoising model: the recent model takes a noisy image and a noise-level map as the input and produces an estimated clean output.

Recent deep learning-based image denoising methods [5, 6] take a noise map and noisy RGB channels as the input. The input goes through carefully designed convolutional layers without pooling or interpolation to directly output clean estimates.  Figure 3 briefly illustrates the models. The performance of deep learning methods has surpassed that of traditional denoising methods including BM3D [3] and NLM [2].

Currently, the noise-level map of deep denoising methods is filled with constant values. However, equation (1) suggests real noise is signal dependent and not homogeneous. Feeding the pixelwise noise-level map with pixelwise estimation is likely to increase the denoising performance.

## 3. Deep Residual Noise-Level Estimation

### 3.1. Problem Formulation

Let the noisy RGB image be denoted by **z** ∈ *R*^*m*×*n*×3^ and the corresponding clean RGB image be denoted by **x** ∈ *R*^*m*×*n*×3^. The image degradation model is written as

(2) $$\begin{array}{c} z=H\left(x\right)=x+n, \end{array}$$

where **n**=[*η*]_*ijk*_ is the noise tensor, which is Gaussian and white. We might as well suppose *η*_*ijk*_ ~ *N*(0, *σ*_*ijk*_) and denote [*σ*]_*ijk*_ as **s**. If we assume noise is not signal dependent, or homogeneous, all *σ*_*ijk*_ should be the same value. Otherwise, *σ*_*ijk*_ might be different values related to image intensity at different pixel locations.

The target of this paper is to learn a dense corresponding mapping function *F*(**z**; *θ*) from the dataset {(**z**_*l*_, **s**_*l*_) | *l*=1,…, *N*} such that

(3) $$\begin{array}{c} \underset{\theta }{\text{min}}\underset{l}{\sum }{\left\|F\left(z_{l};\theta \right)-s_{l}\right\|}_{2}^{2}, \end{array}$$

where *θ* is the model parameter set and *N* is the number of training samples. If we have clean color images **x**_*l*_, the dataset {(**z**_*l*_, **s**_*l*_)} can be easily constructed by randomly setting **s** and applying equation (2). The mapping function *F*(*z*; *θ*) will be implemented by a simple yet carefully designed deep residual network, which we will introduce in Section 3.2.

The goal of this paper is to produce a pixelwise noise-level estimation map, with which we can visualize the noise level over the image. We can also compute a global noise-level scalar from the map and compare the proposed method with traditional estimation methods.

### 3.2. Network Architecture

The 2D convolution samples a regular grid *R* over the input feature map and sums the sampled values with trainable weights [28]. The grid *R* defines the receptive field size and dilation, which ensures the dense correspondence between the input feature map and the output feature map. The correspondence property of convolution makes it good at fitting maps of dense corresponding images.

Fully convolutional networks (FCNs) inherent the correspondence property from 2D convolution, which are good at modeling problems of dense correspondence of images. For example, FCNs are suitable for image segmentation [29] and image denoising [30]. Noise estimation is also a dense correspondence problem, where the output groundtruth noise-level map is densely corresponded with the input noisy image. Therefore, FCNs are expected to have good performance on the problem.

Residual networks [31] are built on the fact that there are difficulties in training deep convolutional neural networks due to gradient vanish. Therefore, if a CNN is too deep, the performance may be worse. Residual networks introduce shortcut connections by simply adding the input to the convolutional output, which mitigates the gradient vanish problem and increases the performance of FCNs.

There are a few layer operations that are not suitable for image denoising. Recent deep denoising methods [5, 6, 30] use network architectures of almost pure convolution layers. They do not use any pooling or interpolation layers since these layers are destructive for image details and will decrease the performance of image reconstruction. Hence, we also abandon pooling or interpolation layers in our network design.

**Figure 4:**
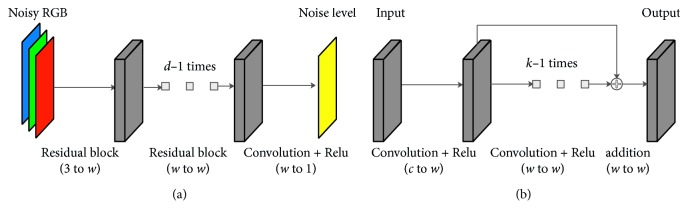
(a) Whole network architecture. The input is a noisy RGB image with three channels, which goes through a stack of residual blocks followed by a convolution and Relu [32] activation. The output is a noise-level channel. (b) Structure of the residual block. Firstly, the *c*-channel input tensor is convolved to a *w*-channel tensor. Then, the *w*-channel tensor goes through a residual structure with *k* − 1 times of convolution and Relu and produces a w-channel output tensor.

A deep residual noise estimation (DRNE) convolutional neural network is proposed.  Figure 4 illustrates the proposed DRNE network architecture.  Figure 4(a) illustrates the whole architecture. The input is a noisy RGB image with three channels, which goes through a stack of residual blocks followed by a convolution and Relu [32] activation. The output is a noise-level channel.  Figure 4(b) illustrates the structure of the residual block. Firstly, the *c*-channel input tensor is convolved to a *w*-channel tensor. Then, the *w*-channel tensor goes through a residual structure with *k* − 1 times of convolution and Relu and produces a *w*-channel output tensor. In the proposed architecture, the total layers of convolutions, or network depth, are *k* × *d*+1.

As for implementation, the sizes of all convolution kernels are 3 × 3. We set the network width and depth by setting *w*=64, *k*=5, and *d*=3, and thus, the total layers of convolutions are 16.

### 3.3. Training

#### 3.3.1. Training Images

Training images are required to be clean and rich textured. We select the first 4,000 of images from the Waterloo exploration dataset [33] to construct training image pairs using (2). Images are cropped into 128 × 128 nonoverlapping patches with a stride of 256.

In theory, every training image needs to be randomly cropped and corrupted with signal-dependent noise levels. However, we find that corrupting every training image with only one noise level in [0,30] to generate training pairs is sufficient to produce satisfactory results. Namely, **s**_*l*_=[*σ*_*l*_]_*ij*_, where *σ*_*l*_ is a scalar uniformly distributed in [0,30].

**Figure 5:**
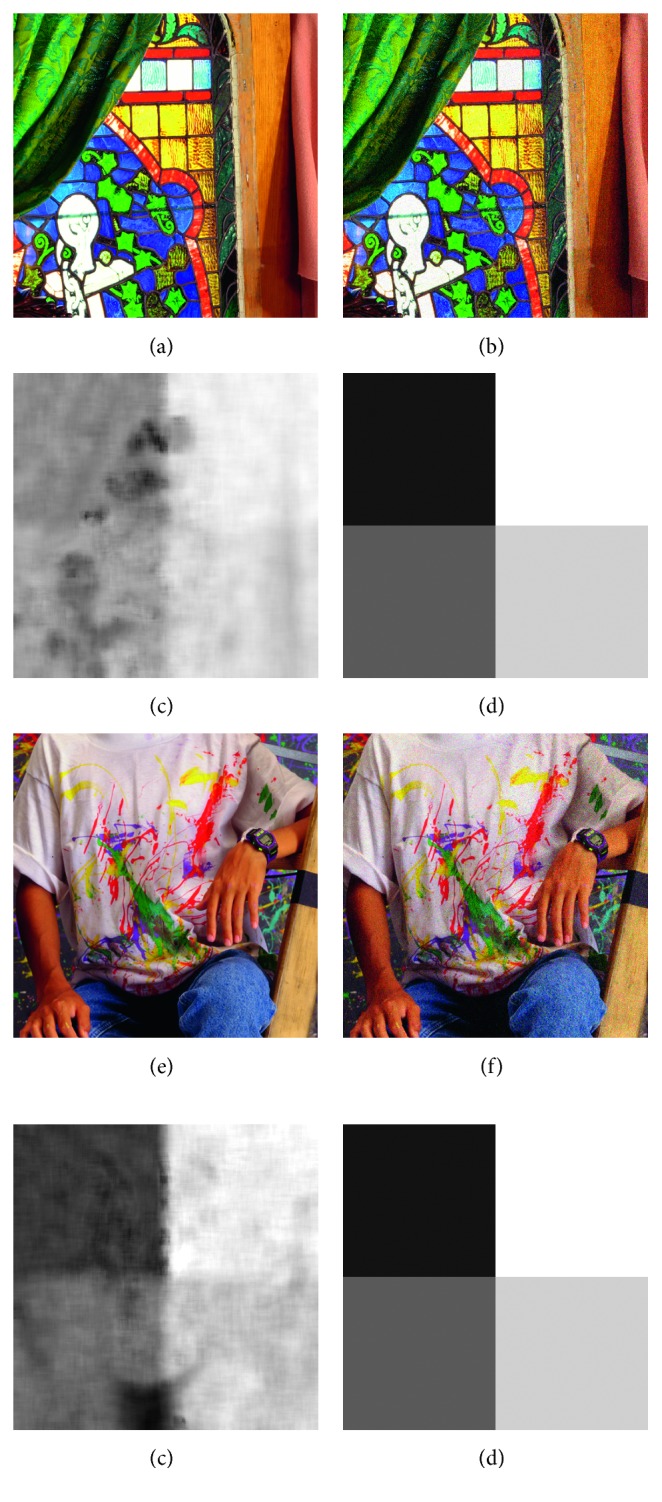
Pixelwise noise-level estimation results. (a, e) Clean images. (b, f) Images with artificial nonhomogeneous Gaussian white noise. The image is divided into four rectangular parts, which are counterclockwise added with noise of levels 1,3,7,  and 9, respectively. (c, g) Estimation results of the proposed noise-level estimation method. (d, h) Groundtruth noise levels for reference.

As a result, our method is trained from homogeneous noisy patches. However, it is able to produce signal-dependent pixelwise noise estimation results in Section 4.3 and Figure 5.

#### 3.3.2. Loss Function

**Figure 6:**
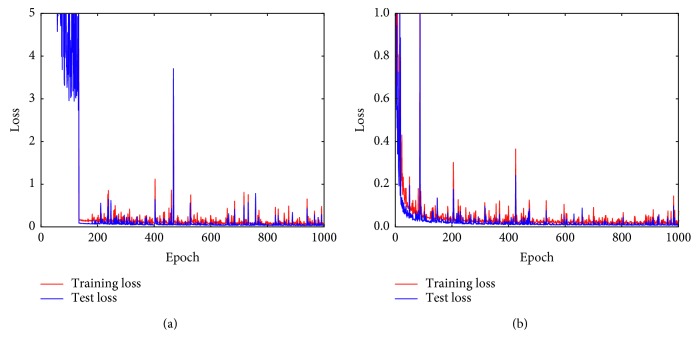
(a) Unregularized loss; (b) regularized loss.

In theory, equation (3) is a reasonable loss function for the task. In practice, however, we find training loss drops with relatively large fluctuations using equation (3).  Figure 6(a) plots the training and validation losses directly using equation (3). In early training epochs, the model is severely underfitting. Later, the losses also suffer from relatively large fluctuations.

The phenomenon suggests the loss function may require regularizations. In the training stage, we feed the model with images corrupted by homogeneous noise. Thus, we use the mean value of elements of the predicted noise matrix to regularize the loss:

(4) $$\begin{array}{c} \underset{\theta }{\text{min}}\underset{l}{\sum }\lambda {\left|\left|F\left(z_{l};\theta \right)-s_{l}\right|\right|}_{2}^{2}+\left(1-\lambda \right)\left|\text{mean}\left(F\left(z_{l};\theta \right)\right)-\sigma_{l}\right|, \end{array}$$

where mean(**x**) is to compute the mean value of elements of the tensor or matrix **x** and *λ* is a scalar parameter to adjust the weights of those two terms.

After applying the regularization term, the training and evaluation losses become significantly more stable and easy to converge.  Figure 6(b) shows the results.

#### 3.3.3. Implementation

We implement the model with TensorFlow [34], the width of which is 64 and the depth is 16, as introduced in Section 3.2. We use equation (4) as the loss function and set *λ*=0.25. The Adam [35] optimizer is adopted for training. It takes around 140 hours to train the model from scratch with Nvidia GTX 1080 Ti GPU.

More ablation study can be done for the parameters. Performance gain may be acquired with intensive trails. Since the experiments in this paper already take a large part, and the current parameter set has good performance in the experiments, we leave it for the future work.

## 4. Experiments

In this section, we give quantitative and qualitative evaluations of the proposed method. In quantitative evaluations, we have to use the traditional scalar average error and standard deviation as evaluation criteria since other methods can only produce scalar estimation. In qualitative evaluations, we visualize the estimation map on both simulated data and real noisy images to show the effectiveness of the proposed method. In addition, we apply comparative methods to two deep denoising methods to reveal the denoising performance gain brought by the DRNE.

### 4.1. Test Datasets and Compared Methods

We compare the proposed method with state-of-the-art methods on three datasets: Kodak, McMaster [36], and BSD500 [37]. Gaussian white noise is added to clean images from the datasets to construct noisy images with groundtruths.

Four methods, including Pyatykh's method [24], Liu's method [25], Chen's method [7], and the proposed DRNE, are compared using their source codes on the same datasets. For all methods to be compared, the parameters of all methods remain unchanged during the comparison.

### 4.2. Comparison on Simulated Homogeneous Noise

We first evaluate all methods on traditional homogeneous noise. The fixed level of homogeneous Gaussian white noise is added to clean RGB images of the three datasets. For a fair comparison, we implement a framework to do noise addition and performance evaluation. Each compared method receives exactly the same input noisy images and uploads estimation results to the framework through a wrapper. Note that Pyatykh's method [24] can only handle gray images. Therefore, noisy RGB images are first transformed to gray images in the wrapper and then processed by Pyatykh's method.

Table 1 shows the average estimation errors of the compared methods on three datasets with six different noise levels. The proposed DRNE won 6 first places and 11 second places. Meanwhile, Chen's method [7] won 12 first places and 3 second places.

Table 2 shows the standard deviation of errors, which implies the stability of the compared methods. The proposed DRNE won 11 first places and 6 second places. Meanwhile, Chen's method [7] won 7 first places and 8 second places.

In general, the performance of Chen's method and the proposed DRNE on homogeneous noise estimation is quite close. The differences are that Chen's method is better at average error, while the proposed DRNE is better at standard deviation.

For example, the average error of Chen's method on the McMaster dataset at noise level 0 is 1.60, which is quite large. More specifically, for the first image of the McMaster dataset shown in Figure 6(a), the estimation result of Chen's method is 4.07, which suggests the method fails to distinguish the difference between high-frequency image details and high-frequency noise. In contrast, the result of the DRNE is 1.42, which is much closer to true noise level 0.

### 4.3. Comparison on Simulated Nonhomogeneous Noise

We then evaluate all methods on nonhomogeneous noise. Clean images from datasets are first divided into four rectangular parts. Then, the four parts are added with noises of different levels: *σ* − 4, *σ* − 2, *σ*+2, and *σ*+4.  Figure 5(b) illustrates the noise addition results, and Figure 5(d) illustrates the groundtruth noise.

Note the noise pattern is designed for easy comparison since it is easy to visualize and compute a global scalar estimation. The real noisy images will be evaluated later.

For traditional methods, the noise estimation results should be the weighted average of noise levels of all patches, which is *σ* in our settings.

Tables 3 and 4 show the average errors and standard deviations of errors, respectively. Similar to the performance on homogeneous noise, Chen's method is better at average error, while the proposed DRNE is better at standard deviation.

The proposed DRNE is not only able to give a scalar prediction but also able to produce a pixelwise noise-level map. Figures 5(c) and 5(g) illustrate the estimated pixelwise noise-level maps, in which the four rectangular parts of different noise levels are obvious. In addition, the estimation results are signal dependent. The flat parts of images tend to be less noisy, which is in accordance with our prior knowledge since it is difficult to distinguish between high-frequency image details and high-frequency noise.

### 4.4. Qualitative Results on Real Images

**Figure 7:**
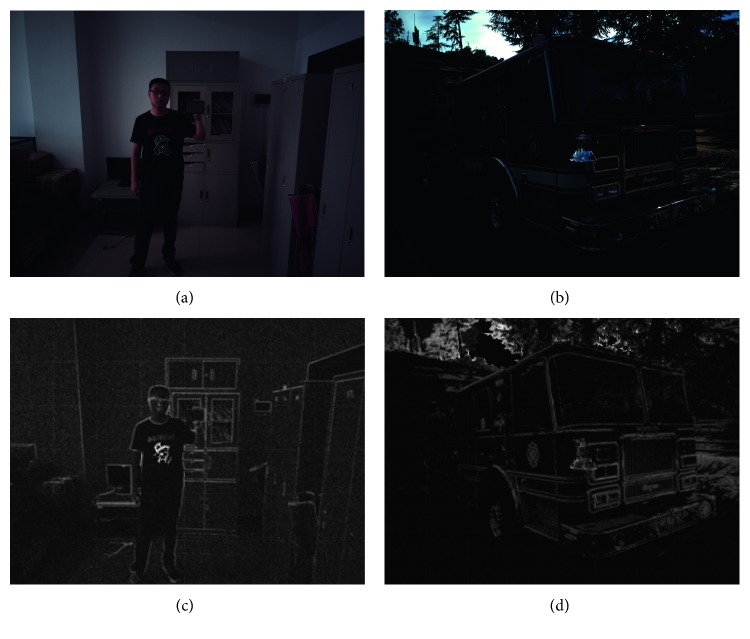
Noise estimation results on a real linear RGB image. In general, the bright part in images suffers from stronger noise, which is in accordance with the noise model (1). The input images are 4K and need to be sliced into patches to process. Therefore, there are some boundary effects in the estimation results. Estimation results are linearly scaled for visualization. (a, b) Real linear RGB image. (c, d) Estimation.

To show the effectiveness of the proposed method on real images, we also evaluate our method on real linear RGB images. These images are captured using mobile phones and saved in raw (DNG) format. Then, they go through early stages of the image processing pipeline without denoising, brightness adjustment, or gamma correction. Figures 7(a) and 7(b) show the processed linear RGB images, and Figures 7(c) and 7(d) show the estimation results of the proposed method.

In general, the dark part in images suffers from weaker noise, which is in accordance with the noise model (1). For example, the black shirt in the image suffered from lower noise levels and the white wall suffered from stronger noise. There are no comparative results of state-of-the-art methods on the task since other methods can only produce a scalar output.

### 4.5. Applying to Deep Learning Denoising

We mentioned in contributions that pixelwise noise estimation is expected to improve the performance on deep learning denoising methods [5]. Now, we design an experiment to validate the conclusion.

First, we prepare two existing deep denoising models [6, 38] which take noisy images and noise-level maps as the input. Then, homogeneous and nonhomogeneous noisy images are generated using different strategies. At last, we use Chen's method and the proposed method to feed the noise map to the deep denoising model. Denoising performance is recorded.

Previous evaluations have shown that the performance of Chen's method [7] and the proposed method is close, while that of other comparative methods significantly surpassed. Therefore, we focus on comparison with Chen's method.

Table 5 demonstrates the denoising performance on homogeneous noise. The performance on combination of three datasets and three noise levels is reported. We can see that when dealing with homogeneous noise, the performance of Chen's method and the proposed DRNE is comparable, while the proposed DRNE works slightly better in most test cases. We can conclude from Table 5 that the DRNE is an alternative to Chen's method in removing homogeneous noise.

Table 6 demonstrates the denoising performance on nonhomogeneous noise. Two noise models are tested: (1) Noise variance *σ* is uniformly distributed in a fixed range. The performance of the two methods is also close. The DRNE works slightly better in most test cases. (2) Noise variance follows noise model equation (1) with *σ*_*r*_ ~ *U*(0,15) and *σ*_*s*_ ~ *U*(0,3.5). We clip the noise variance to ensure it does not exceed the prediction range of the DRNE.

When the real noise model is adopted, the DRNE got a significant performance gain on Kodak and BSD500 datasets. On the McMaster dataset, the performance of the two methods is close. We can conclude from Table 6 that the DRNE shows generally better performance in removing nonhomogeneous noise when dealing with real noise.

**Figure 8:**
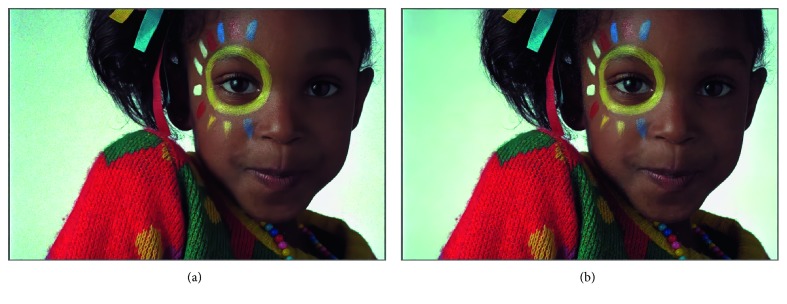
Applying noise estimation methods to deep denoising of nonhomogeneous noise. (a, b) Visual results of the test case noise model (1) on the Kodak dataset from Table 6. We can see obvious artifacts in the background part of (a), while (b) shows no artifacts.

Figure 8 shows visual results of noise model (1) on the Kodak dataset from Table 6. We can see obvious artifacts in the background part in Figure 8(a), while Figure 8(b) shows no artifacts, which suggests that Chen's method does not adapt well to the more realistic noise model and shows the effectiveness of the proposed DRNE.

In this section, we show that when working with the CNN denoising model, the proposed DRNE is generally better in removing nonhomogeneous noise with both quantitative and qualitative results.

### 4.6. Running Time Comparison

In order to handle large images, the DRNE crops images into patches with the fixed size and handles them sequentially. For a fair comparison, we use the McMaster dataset for evaluation and set the crop size the same as the input image size 500 × 500.

Table 1 shows the running time comparison of the compared methods on datasets, which is a measure on a desktop with Intel i7-5930K CPU and Nvidia GTX 1080 Ti GPU. The DRNE is implemented with Python, and other methods are implemented with MATLAB. Chen's method is the fastest on CPU. The DRNE is the slowest on CPU partially because matrix computation in Python is not heavily optimized as in MATLAB. However, the DRNE is the second fastest with the help of GPU.

## 5. Conclusion

In this paper, we propose a deep residual convolutional neural network named “DRNE” for Gaussian noise-level map estimation of images. The main architecture consists of a stack of carefully designed customized residual blocks. Given a noisy image, the proposed DRNE is able to produce a pixelwise noise-level estimation map as well as an overall scalar noise level.

Experiments show that the proposed DRNE is able to compete with state-of-the-art methods such as Chen's method on traditional scalar noise-level estimation. In addition, the DRNE is able to produce a signal-dependent noise-level map, which is in accordance with the linear RGB image noise model (1).

Pixelwise noise-level estimation is helpful for precise noise removal. Recent deep learning-based noise removal methods [5, 6] require a pixelwise noise-level estimation map as the input for noise removal. By applying the DRNE and Chen's method to deep learning denoising models, we reveal that the DRNE can bring significant performance gains in removing signal-dependent Gaussian noise (more close to real noise than traditional Gaussian noise with fixed variance).

Deep learning might be the ultimate method to separate high-frequency image details from noise by automatically mining patterns from image data. For the future work, we believe that joint training of the deep noise estimation model and the deep denoising model is possible to surpass all traditional methods in image denoising.

## Figures and Tables

**Table 1:** Average error on simulated homogeneous noise (dB).

Dataset	Noise level	Pyatykh [24]	Liu [25]	Chen [7]	DRNE
Kodak (24 images)	*σ*=0	0.91	0.96	**0.68**	*0.74*
	*σ*=5	1.79	0.17	*0.09*	**0.08**
	*σ*=10	3.93	0.19	**0.12**	*0.18*
	*σ*=15	6.13	0.33	**0.29**	**0.29**
	*σ*=20	8.42	0.55	*0.52*	**0.51**
	*σ*=25	10.87	0.90	*0.85*	**0.80**

McMaster (18 images)	*σ*=0	*0.62*	**0.49**	1.60	0.83
	*σ*=5	1.70	*0.16*	0.23	**0.11**
	*σ*=10	4.12	0.65	**0.32**	*0.41*
	*σ*=15	6.93	1.22	**0.81**	*0.91*
	*σ*=20	9.84	1.89	**1.51**	*1.53*
	*σ*=25	12.73	2.65	**2.17**	*2.26*

BSD500 (500 images)	*σ*=0	0.52	*0.35*	0.54	**0.32**
	*σ*=5	1.66	0.18	**0.05**	*0.13*
	*σ*=10	3.70	0.35	**0.21**	*0.32*
	*σ*=15	5.93	0.57	**0.46**	*0.53*
	*σ*=20	8.30	0.90	**0.79**	*0.85*
	*σ*=25	10.79	1.33	**1.23**	*1.26*

Bold fonts denote the best performance and italics denotes the second best performance.

**Table 2:** Standard deviation of error on simulated homogeneous noise (dB).

Dataset	Noise level	Pyatykh [24]	Liu [25]	Chen [7]	DRNE
Kodak (24 images)	*σ*=0	0.45	0.30	*0.22*	**0.09**
	*σ*=5	0.51	0.26	**0.15**	**0.15**
	*σ*=10	1.00	0.49	*0.36*	**0.30**
	*σ*=15	1.46	0.67	*0.55*	**0.50**
	*σ*=20	1.91	0.86	*0.75*	**0.71**
	*σ*=25	2.31	1.07	**0.95**	*0.96*

McMaster (18 images)	*σ*=0	*0.16*	**0.10**	0.71	0.24
	*σ*=5	*0.17*	0.18	0.28	**0.11**
	*σ*=10	0.52	0.46	*0.34*	**0.32**
	*σ*=15	0.87	0.76	*0.67*	**0.62**
	*σ*=20	1.11	1.09	*1.03*	**0.94**
	*σ*=25	*1.35*	1.42	1.37	**1.28**

BSD500 (500 images)	*σ*=0	0.77	0.26	*0.21*	**0.07**
	*σ*=5	1.12	1.31	**0.08**	*0.11*
	*σ*=10	0.80	0.42	**0.22**	*0.26*
	*σ*=15	1.25	0.61	**0.41**	*0.47*
	*σ*=20	1.71	0.85	**0.64**	*0.72*
	*σ*=25	2.17	1.11	**0.91**	*1.00*

Bold fonts denote the best performance and italics denotes the second best performance.

**Table 3:** Average error on simulated nonhomogeneous noise (dB).

Dataset	Noise level	Pyatykh [24]	Liu [25]	Chen [7]	DRNE
Kodak	*σ* ~ *U*(1,9)	3.33	1.08	*0.91*	**0.07**
	*σ* ~ *U*(11,19)	7.34	0.48	**0.30**	*0.31*
	*σ* ~ *U*(21,29)	11.71	0.86	**0.67**	*0.84*

McMaster	*σ* ~ *U*(1,9)	3.39	1.18	1.03	**0.12**
	*σ* ~ *U*(11,19)	7.88	1.46	**0.56**	*0.91*
	*σ* ~ *U*(21,29)	13.31	2.74	**2.01**	*2.27*

BSD500	*σ* ~ *U*(1,9)	3.15	1.47	0.82	**0.23**
	*σ* ~ *U*(11,19)	7.12	0.81	**0.31**	*0.58*
	*σ* ~ *U*(21,29)	11.56	1.38	**1.08**	*1.32*

Bold fonts denote the best performance and italics denotes the second best performance.

**Table 4:** Standard deviation of error on simulated nonhomogeneous noise (dB).

Dataset	Noise level	Pyatykh [24]	Liu [25]	Chen [7]	DRNE
Kodak	*σ* ~ *U*(1,9)	1.67	0.64	*0.19*	**0.16**
	*σ* ~ *U*(11,19)	1.40	0.68	*0.54*	**0.52**
	*σ* ~ *U*(21,29)	2.45	1.10	*1.05*	**0.97**

McMaster	*σ* ~ *U*(1,9)	0.61	0.70	*0.31*	**0.10**
	*σ* ~ *U*(11,19)	0.76	1.03	**0.55**	*0.63*
	*σ* ~ *U*(21,29)	*1.31*	1.58	1.35	**1.28**

BSD500	*σ* ~ *U*(1,9)	1.03	0.86	0.14	**0.12**
	*σ* ~ *U*(11,19)	1.11	0.77	**0.37**	*0.48*
	*σ* ~ *U*(21,29)	1.94	1.20	**0.91**	*1.00*

Bold fonts denote the best performance and italics denotes the second best performance.

**Table 5:** Applying noise estimation methods to deep learning models to remove homogeneous noise (dB).

Dataset	Noise level	Chen [7] + DRDD	DRNE + DRDD	Chen + FFDNet	DRNE + FFDNet
Kodak	*σ*=5	**39.87**	**39.87**	39.80	39.80
	*σ*=15	34.84	**34.85**	34.84	**34.85**
	*σ*=25	32.99	33.02	33.04	**33.06**

McMaster	*σ*=5	**39.17**	39.15	38.84	38.84
	*σ*=15	34.74	**34.75**	34.65	34.67
	*σ*=25	32.26	**32.36**	32.34	32.42

BSD500	*σ*=5	**39.36**	39.35	39.36	39.35
	*σ*=15	34.26	**34.27**	34.23	34.24
	*σ*=25	32.34	32.39	32.36	**32.40**

**Table 6:** Applying noise estimation methods to deep learning models to remove nonhomogeneous noise (dB).

Dataset	Noise level	Chen [7] + DRDD	DRNE + DRDD	Chen + FFDNet	DRNE + FFDNet
Kodak	*σ* ~ *U*(1,9)	**39.91**	39.90	39.84	39.84
	*σ* ~ *U*(11,19)	34.97	**34.98**	34.97	**34.98**
	*σ* ~ *U*(21,29)	33.04	33.07	33.10	**33.12**
	*σ* ~ *U*(1,29)	36.29	36.31	36.38	**36.40**
	Noise model (1)	33.30	*33.63*	33.24	*33.68*

McMaster	*σ* ~ *U*(1,9)	39.62	**39.63**	39.30	39.32
	*σ* ~ *U*(11,19)	35.04	**35.05**	34.93	34.95
	*σ* ~ *U*(21,29)	32.30	32.39	32.38	**32.45**
	*σ* ~ *U*(1,29)	34.66	**34.72**	34.61	34.67
	Noise model (1)	**33.21**	33.18	33.14	33.14

BSD500	*σ* ~ *U*(1,9)	**39.47**	39.46	39.44	39.43
	*σ* ~ *U*(11,19)	34.28	**34.29**	34.25	34.26
	*σ* ~ *U*(21,29)	32.34	32.38	32.35	**32.40**
	*σ* ~ *U*(1,29)	35.66	**35.67**	35.64	35.66
	Noise model (1)	33.01	*33.20*	32.92	33.17

Text in bold denotes the best method. Text in italics denotes significant performance gains of the DRNE against Chen's method.

**Table 7:** Average running time (seconds).

Dataset	Pyatykh (CPU)	Liu (CPU)	Chen (CPU)	DRNE (CPU/GPU)
McMaster	1.75	2.20	**0.27**	5.31/1.16

## Data Availability

The three datasets Kodak, McMaster, and BSD500 are publicly available at http://www.cs.albany.edu/∼xypan/research/snr/Kodak.html, http://www4.comp.polyu.edu.hk/∼cslzhang/CDM_Dataset.htm, and https://www2.eecs.berkeley.edu/Research/Projects/CS/vision/bsds/, respectively. We also provide code and pretrained models to reproduce the experimental results in our paper at https://github.com/TomHeaven/Pixel-wise-Estimation-of-Signal-Dependent-Image-Noise-using-Deep-ResidualLearning.
